# Quantification of Dysautonomia in Major Depressive Disorder Using the Composite Autonomic Scoring Scale-31 (COMPASS-31)

**DOI:** 10.7759/cureus.48008

**Published:** 2023-10-30

**Authors:** Sumit Kumar, Arijita Banerjee

**Affiliations:** 1 Psychiatry and Behavioral Sciences, Tata Main Hospital, Jamshedpur, IND; 2 Physiology, Indian Institute of Technology, Kharagpur, Kharagpur, IND

**Keywords:** screening tool, orthostatic intolerance, vasomotor symptom, depression, dysautonomia

## Abstract

Background

Dysautonomia denotes an alteration in the autonomic nervous system that can significantly lead to multiorgan failure. Conversely, depression not only affects cognitive functions but also poses a risk factor for sudden cardiac death. In our study, the Composite Autonomic Scoring-31 (COMPASS-31) is used to quantify the autonomic symptoms present if any in patients with depression.

Materials and methods

Forty-two patients with major depressive disorder (MDD) were recruited using a PHQ-9 questionnaire followed by a COMPASS-31 scale to quantify dysautonomia symptoms, and they were compared with healthy controls. Further regression analysis was conducted to establish any relationship between independent variables and COMPASS-31 scores.

Results

The average COMPASS-31 score in patients with MDD was 22.56±8.42, which was significantly increased compared to healthy controls (p=0.001). Furthermore, the differences persisted across various subdomains of the COMPASS-31 scale relative to severity of depression.

Conclusion

The study observations could provide a relevant perception regarding the association between depression and autonomic dysfunction with the use of a simple and brief yet validated instrument COMPASS-31, which can be utilized for screening at the primary care level.

## Introduction

Depression is one of the prevailing mental disorders that adversely influences emotional and cognitive functions, affecting people globally with a prevalence rate of 2.9% [1]. Moreover, depressive disorder poses a determinant with a high risk of developing cardiovascular disorder (CVD), leading to sudden cardiac death. However, only a few studies explore the link between depression and cardiovascular health [2,3]. Dysautonomia is defined as an alteration in the autonomic nervous system that can significantly affect a person’s health. Sympathovagal imbalance is frequently related to an increased risk of sudden cardiopulmonary arrest. Heart rate variability (HRV) and other cardiovascular reflex tests (CART) are sensitive, quantitative, and noninvasive tools, which suggests the dynamicity of the autonomic nervous system [4,5].

The kynurenine metabolic pathway is affected by immune dysregulation, and the latter is considered to play an important model in the pathophysiological mechanisms of depression [6]. Simultaneously, immune dysregulation is also considered to play a role in accelerated atherosclerosis [7]. Thus, immune dysregulation could affect both mental and cardiovascular health, contributing to depression being called a “neurocardiovascular” disorder. In this regard, a symptoms-based questionnaire assessing autonomic dysfunction in patients with depression could be a promising screening tool at the primary care level. The Composite Autonomic Scoring Scale (COMPASS)-31 is an authenticated tool, consisting of 31 questions, a brief version of the exhaustive 164-item COMPASS assessment questionnaire used to quantify autonomic failure in autonomic neuropathy patients [8].

In the current study, we used a COMPASS-31 scale to quantify the autonomic symptoms present if any in patients with major depressive disorder (MDD).

## Materials and methods

A cross-sectional study was conducted between January 2023 and September 2023 in patients with major depressive disorder. All subjects were recruited from the Psychiatry Outpatient Department of Dr B.C. Roy Multi-Speciality Medical Research Centre, Indian Institute of Technology, Kharagpur, and Tata Main Hospital, Jamshedpur, India.

Ethical Considerations

The study is part of a larger research project approved by the Institutional Ethics Committee Board (IIT/SRIC/DEAN/2023). Informed consent was obtained from all participants belonging to the study sample.

Sample Size

Considering the prevalence of depression in individuals above 18 years to be 5.25% in India as reported by Arvind et al. in 2019, the formula used to calculate sample size was Z2 PQ / M2, with a 99% confidence interval (CI) [9]. In this way, by purposive sampling, we finally recruited 42 patients with depression and 42 healthy controls for our study.

Inclusion Criteria

The study subjects of age between 18 and 45 years of either sex were included in the study to avoid the autonomic function changes with increasing age. A total of 42 patients were selected with the diagnosis of MDD by a trained psychiatrist according to a Patient Health Questionnaire (PHQ-9) score ≥10. A total of 42 healthy controls were matched regarding age and gender. All healthy controls had a PHQ-9 score <5 points, with the absence of any individual or family history of mental illness. Subjects with scores ranging between 5 and 9 were asked for a regular follow-up.

Participants with a history of any of these like chronic metabolic disorder, history of substance abuse, neurological disorder, cardiovascular disorders, gastrointestinal problems, and pregnant females were excluded from the study.

Aim of the Study

The study was done to compare COMPASS-31 scores in patients with MDD with healthy controls.

Depression Severity Assessment

The Patient Health Questionnaire (PHQ) is a validated tool for the diagnosis of depression and its severity in primary care. It is a nine-item questionnaire with scores ranging from 0 to 27 and with scores between 10 to 27, indicative of major depression (MDD). The scale has an internal validity of 0.89 with an ROC analysis in diagnosing major depression to be 0.95 [10].

COMPASS-31

This scale includes 31 questions pertaining to mainly six domains of sympathovagal imbalance such as gastrointestinal dysfunction, vasomotor imbalance, secretomotor disturbances, pupillomotor abnormalities, orthostatic intolerance, and urinary dysfunction. The total scoring if added, usually ranges between zero and 100, with a maximum score of 40 for orthostatic intolerance followed by 25 for gastrointestinal dysfunction, 15 for glandular abnormalities, and 10 for urinary ailments with five each for vasomotor and pupillomotor problems, respectively. The scale has an outstanding internal validity of 0.9 [11].

Data Analysis

Sociodemographic information comprising age, body mass index (BMI), gender, spousal status, educational standards, employment, and duration of depression were recorded. Data analysis was conducted using Statistical Product and Service Solutions (SPSS) (version 22.0; SPSS Statistics for Windows, Armonk, NY), and the comparison of COMPASS-31 scores between two groups was assessed using an unpaired “t” test for normally distributed data; otherwise, a Wilcoxon rank-sum test was used for data that were not normally distributed. Regression analysis was performed to establish any relationship between independent variables and COMPASS-31 scores.

## Results

Eighty-four subjects were asked to complete the COMPASS-31 questionnaire. Table 1 depicts the vital statistics of study participants’ demographic information. The present study comprised 42 patients with depression where the mean age was 31.04±8.42 years and 42 healthy controls where the mean age was 29.12±8.44 years, both of which were not statistically significant. Sixty-seven percent (28) of patients and 62% (26) of healthy controls were male. The mean disease duration from the onset of symptoms of depression among the patients was 3.02±1.76 years. The majority of the patients (71%) and subjects (64%) were educated and belonged to middle-class family status. However, the incidence of irregular medication among the patients with depression in our study was estimated to be 19%. The mean BMI of the patients was significantly raised than normal (26.14±7.2), which makes them fall under the obese category (p=0.001). No significant differences were observed in other socioeconomic parameters between the two groups.

**Table 1:** Sociodemographic Characteristics of the 84 Subjects MDD-Major Depressive Disorder; SD-Standard Deviation; BP-Blood Pressure

Characteristics	Numbers (N) (Total=84) MDD (42)	Controls (42)
	N/Mean±SD	N/Mean±SD
Mean age (years)	31.04±8.42 (p=0.14)	29.12±8.44
2. Gender		
Male	28	26
Female	14	16
3. Marital Status		
Married	17	20
Widow/widower	0	0
unmarried	25	22
4. Education		
Primary School	6	4
High School	10	8
Secondary School	26	30
5. Socioeconomic class (Kuppuswamy)		
Middle-III	30	27
Lower middle-IV	12	15
6. Employed	21	28
7. Body mass index (mean)	26.14±7.2 (p=0.001)	22.67±3.8
8. Systolic BP (mmHg)	124.32±7.84 (p=0.001)	120.56±10.42
Diastolic BP (mmHg	82.44±10.34 (p=0.002)	78.66±7.83
9. Mean duration of depression (years)	3.02 ±1.76	0
10. Regular in medication/drugs	34	0

**Figure 1:**
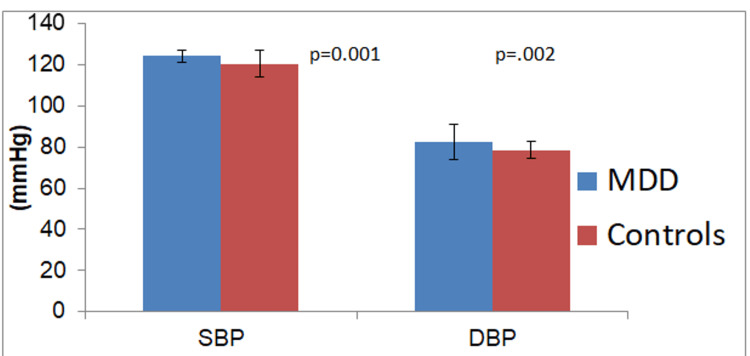
Mean Systolic Blood Pressure and Diastolic Blood Pressure SBP-Systolic Blood Pressure; DBP-Diastolic Blood Pressure; MDD-Major Depressive Disorder

**Figure 2:**
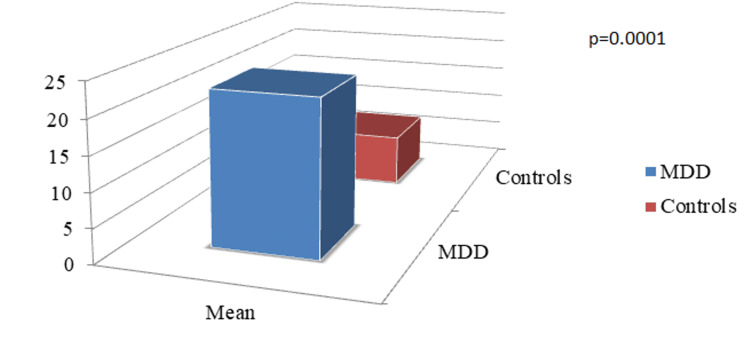
Comparison of Mean COMPASS-31 Scores MDD-Major Depressive Disorder

**Figure 3:**
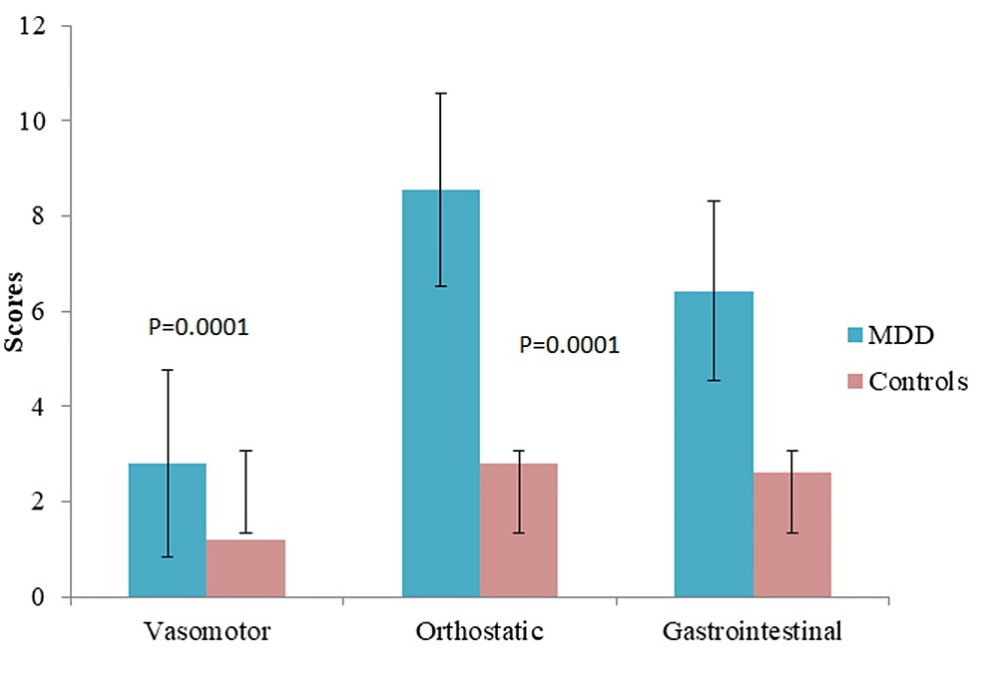
Comparison of Scores of Subdomains of COMPASS-31 MDD-Major Depressive Disorder

**Figure 4:**
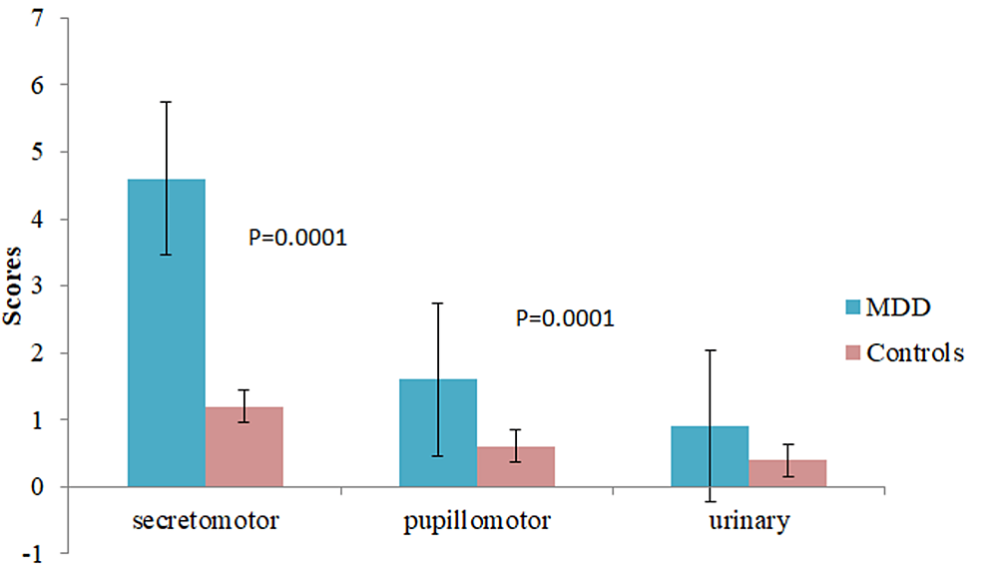
Comparison of Scores of Subdomains of COMPASS-31 MDD-Major Depressive Disorder

The mean systolic blood pressure (SBP) and diastolic blood pressure (DBP) among the patients with MDD were 124.32±7.84 mmHg and 82.44±10.34 mmHg, respectively, which are significantly different (p=0.001) from the controls (Figure 1). The average COMPASS-31 score in patients with MDD was 22.56±8.42. It was documented from the study that subjects with MDD had significantly increased COMPASS-31 scores in contrast to healthy controls (7.6±2.72, p=0.0001). Moreover, various subdomains of COMPASS-31 scores showed significant differences when compared with healthy controls, and the highest score was obtained in the orthostatic domain (8.54±3.22, p=0.0001) followed by the gastrointestinal domain (6.42±2.84, p=0.002), as shown in Figures 2-4.

On performing linear regression analysis between various variables and mean COMPASS-31 scores, it was found that patients with moderate MDD (scores between 10 and 14) had significantly increased mean COMPASS-31 scores compared to no or minimal depression subjects. Similarly, patients with moderately severe MDD (scores between 15-19) and patients with severe MDD (scores between 20-27) also had significantly higher mean COMPASS-31 scores compared to no or minimal depression subjects. All the above differences were constant even after adjusting for age and sex (3.76 (1.48-8.67), p=0.004). While some patients were obese or overweight or hypertensive, they had significantly raised COMPASS-31 scores compared to nonobese patients or normotensives; however, the difference was lost after adjusting for age and sex (1.12(0.64-2.33), p=0.18). On the other hand, patients with MDD who had irregular medications like antidepressant drugs had increased COMPASS-31 scores that remained significant even after the adjustment (2.48 (1.42-4.28), p=0.005), as shown in Table 2.

**Table 2:** Multivariate Linear Regression Analysis Between Dependent Variables and Mean COMPASS-31 Scores MDD-Major Depressive Disorder; OR-Odds Ratio; CI-Confidence Interval; BMI-Body Mass Index

Dependant variables	Unadjusted OR (95% CI)	P value	Adjusted with age and sex OR (95% CI)	P value
MDD severity (scores from 0 to 4) - reference				
10-14 (moderate)	6.42 (2.69–10.34)	<0.001	3.76 (1.48–8.67)	0.004
15-19 (moderately severe)	3.44 (1.78- 9.24)	<0.001	1.82 (0.72- 2.86)	0.002
20-27 (severe)	1.06 (0.88–2.67)	0.002	0.82 (0.64–2.14)	0.05
BMI (ref. normal ≤22.9)				
Overweight or obese (≥23)	1.86 (0.98-3.14)	0.02	1.12 (0.64-2.33)	0.18
Regularity in taking antidepressants (ref. regular)				
Irregular	3.12 (1.68-4.66)	0.001	2.48 (1.42-4.28)	0.005
Mean arterial pressure (ref: normal < 92 mmHg)	6.24 (2.78–7.67)	0.005	4.82 (2.56–6.04)	0.08

## Discussion

The present study was conducted to assess and quantify dysautonomia in patients with MDD using a validated screening tool, the COMPASS-31 questionnaire. To date, few studies have conducted research analysis on various pathophysiological mechanisms of depression, but autonomic dysregulation in depression remains a topic of debate [12,13]. So far, there has been no gold standard test for the quantification of symptoms of autonomic dysregulation. In practice, noninvasive autonomic function tests along with heart rate variability are usually used to address the sympathovagal imbalance in various categories of patients. While the mentioned tests have proved to be effective, the expensive nature of the tests and the required expertise have emphasized the need for an established screening instrument to quantify symptoms of autonomic dysregulation [14].

In accordance with previous studies, it was found that patients with MDD have an increased risk of autonomic failure. At the primary care level, blood pressure measurement and resting heart rate do not predict the implications of autonomic dysfunction in depression; thus, our study contributes to the use of COMPASS-31 questionnaire scores in screening depression patients for dysautonomia [15].

In the present study, patients with MDD were considered to have significant orthostatic intolerance and vasomotor disturbances, indicative of global dysautonomia. These findings are consistent with previous studies that evaluated the associations between orthostatic intolerance and depression using an orthostatic intolerance questionnaire [16]. Moreover, gastrointestinal disturbances were significantly noted in the depressive subjects, raising various questions on brain-gut interplay. Earlier, a study in 2016 suggested the direct stimulation of the vagus nerve, which is the main connecting link in the gut-brain axis, leading to anxiety-like behavior in mice. In accordance with the above findings, probiotics have been explored and utilized as an adjuvant therapy for various psychiatric disorders [17].

From our study, it is observed that global autonomic dysfunction was observed among patients with MDD. The findings indicative of sudomotor dysfunction were in accordance with prior studies, which suggested that because of the neurogenic inflammation of the dense autonomic fibers in various disorders involving the central nervous system, sudomotor control is lost, resulting in an imbalance in thermoregulation usually detected by either quantitative sudomotor axon reflex testing or thermoregulatory sweat testing [18].

Moreover, in our study, pupillomotor dysfunction was observed in patients with MDD. Pupillomotor function has been considered as lost gradually in various disorders involving the peripheral nervous system and central nervous system like Alzheimer’s disease and Parkinsonism. Further studies are required using dynamic pupillometry for a quantitative evaluation of light reflex in these patients [19]. The findings of urinary dysfunction were also in accordance with previous studies that mentioned that urinary incontinence and lower urinary tract infections are prevalent in patients with depression. The statistical significance in differences among the scores spanning various stages of depression could suggest unexplored yet important pathophysiological mechanisms of dysautonomia in depression [20].

The robustness of our study comprises the application of a validated questionnaire that assesses global dysautonomia rather than a single aspect of the autonomic nervous system [21]. However, the COMPASS-31 questionnaire measures the existence of dysautonomia rather than highlighting the extent of sympathovagal imbalance. It is recommended to pursue qualitative assessment using various reflex tests, spectral analysis of Holter monitoring and baroreflex sensitivity, and the identification of potential biomarkers for early detection of dysautonomia in patients with MDD after initial screening with the COMPASS-31 scale.

The study limitations include the lack of the use of qualitative tests for autonomic dysfunction and the correlation of the same with the COMPASS-31 scale, forming the basis for future research. Moreover, the antidepressants used by the patients with MDD were not categorized separately as most of them were given as combination therapy using selective serotonin reuptake inhibitors and tricyclic antidepressants.

## Conclusions

The study observations have provided relevant perceptions about the association between low mood and various domains of dysautonomia in patients with MDD. Future research studies investigating the pathophysiological mechanisms of dysautonomia in depression are warranted. COMPASS-31, a self-administered, verified scale to quantify symptoms of autonomic imbalance can be used by physicians at the primary care level as a screening tool in patients with depression so that treatments for the latter should target the nervous system with an emphasis on sympathovagal dynamics.
